# Janus Type Monolayers of S-MoSiN_2_ Family and Van Der Waals Heterostructures with Graphene: DFT-Based Study

**DOI:** 10.3390/nano12213904

**Published:** 2022-11-05

**Authors:** Ruslan M. Meftakhutdinov, Renat T. Sibatov

**Affiliations:** 1 Laboratory of Diffusion Processes, Ulyanovsk State University, 432017 Ulyanovsk, Russia; 2 Scientific-Manufacturing Complex “Technological Centre”, 124498 Moscow, Russia

**Keywords:** Janus 2D material, transition metal dichalcogenide, van der Waals heterostructure

## Abstract

Novel representative 2D materials of the Janus type family X-M-ZN${}_{2}$ are studied. These materials are hybrids of a transition metal dichalcogenide and a material from the MoSi${}_{2}$N${}_{4}$ family, and they were constructed and optimized from the MoSi${}_{2}$N${}_{4}$ monolayer by the substitution of SiN${}_{2}$ group on one side by chalcogen atoms (sulfur, selenium, or tellurium), and possibly replacing molybdenum (Mo) to tungsten (W) and/or silicon (Si) to germanium (Ge). The stability of novel materials is evaluated by calculating phonon spectra and binding energies. Mechanical, electronic, and optical characteristics are calculated by methods based on the density functional theory. All considered 2D materials are semiconductors with a substantial bandgap (>1 eV). The mirror symmetry breaking is the cause of a significant built-in electric field and intrinsic dipole moment. The spin–orbit coupling (SOC) is estimated by calculations of SOC polarized bandstructures for four most stable X-M-ZN${}_{2}$ structures. The possible van der Waals heterostructures of considered Janus type monolayers with graphene are constructed and optimized. It is demonstrated that monolayers can serve as outer plates in conducting layers (with graphene) for shielding a constant external electric field.

## 1. Introduction

Recently [1], centimeter-scale monolayer films of 2D material MoSi${}_{2}$N${}_{4}$ were obtained by introducing elemental silicon during growth of non-layered molybdenum nitride by chemical vapor deposition. Successful formation of analogous 2D material WSi${}_{2}$N${}_{4}$ with a similar approach was evidence of the concept versatility. The method opened up opportunities for elaboration of a family of stable 2D materials for which there are no natural layered 3D crystals [2].

Two-dimensional MoSi${}_{2}$N${}_{4}$ consists of a MoN${}_{2}$ monolayer sandwiched between two Si-N layers. MoSi${}_{2}$N${}_{4}$ demonstrates semiconducting behavior with excellent ambient air stability. MoSi${}_{2}$N${}_{4}$ is characterized by an elastic constant three times higher than a constant of 2D transition metal dichalcogenide MoS${}_{2}$. The electron and hole mobilities in MoSi${}_{2}$N${}_{4}$ are approximately 4 and 6 times higher than for MoS${}_{2}$. High carrier mobility coupled with high environmental stability makes this material promising for use in various nanoelectronic applications.

A large number of similar 2D materials with general formula MA${}_{2}$Z${}_{4}$ (M = Mo, W, V, Nb, Ta, Ti, Zr, Hf or Cr; A = Si or Ge, Z = N, P or As) were predicted by means of the density functional theory (DFT) based computations and investigated using computational methods (see, e.g., [3,4]). The predicted monolayers are characterized by a wide range of properties from semiconductor to metallic; some compounds with magnetic transition metal elements also have magnetic properties [5]. MoSi${}_{2}$N${}_{4}$ and WSi${}_{2}$N${}_{4}$ monolayers also exhibit high lattice thermal conductivity for thermoelectric applications [6]. Most of the 2D materials from the discussed family also have a suitable band gap of up to 1.7 eV for potential optical applications in the visible range [7,8].

In our recent work [9], for the first time, we proposed and theoretically investigated the asymmetric 2D materials XMoSiN${}_{2}$ (X = S, Se, Te), constructed and optimized from MoSi${}_{2}$N${}_{4}$ by substitution of SiN${}_{2}$ group on one side of MoSi${}_{2}$N${}_{4}$ by chalcogen atoms (sulfur, selenium, or tellurium). The constructed 2D materials are hybrids of a transition metal dichalcogenide and a 2D material from the MoSi${}_{2}$N${}_{4}$ family. They can be considered as a Janus type 2D material with mirror symmetry breaking. Hypothetically, the experimental methods of chalcogen replacement in conventional 2D transition-metal dichalcogenides to form Janus-type heterostructures (see, e.g., [10,11,12]) can be conformed to obtain the proposed layers.

**Figure 1:**
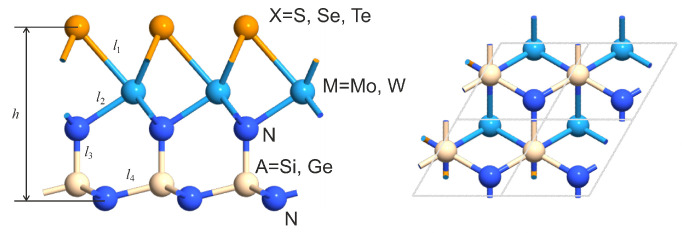
Geometry of the XMAN${}_{2}$ monolayer. Lattice parameter, bond lengths, and thickness are listed in Table 1.

The geometry of the structures under consideration is presented in Figure 1. Parameters of novel optimized structures are provided in Section 2. The stability is substantiated by DFT-based calculations of phonon bandstructures and binding energies. The mirror symmetry breaking and significant difference in electronegativity of chalcogen atoms and an SiN${}_{2}$ group lead to high intrinsic electric field. A large built-in transverse electric field enables separation of the generated electron–hole pairs within a monolayer that is promising particularly for photovoltaic applications.

Recently, Rezavand and Ghobadi [13] studied the structural, electronic, and spintronic features of similar Janus MTeSiX${}_{2}$ (M = Mo, W; X = N, P, As) monolayers by computational methods. Calculated phonon bandstructures and cohesive energies indicate the stability of these Janus monolayers. The considered Janus type monolayers demonstrate a semiconducting behavior with a sufficient bandgap. The authors confirmed a high intrinsic electric field caused by breaking out-of-plane symmetry. This field induces Rashba spin–orbit coupling. In addition, the authors observed non-parabolic Mexican hat-like dispersion at the Γ-point of the valence band. Studies [9,13] enriched a Janus 2D materials family by novel candidates for next-generation spintronic or photovoltaic devices.

In this paper, we supplement the family of asymmetric structures X-M-ZN${}_{2}$ by novel representative 2D materials (namely, X = S, Se, Te; M = Mo, W; A = Si, Ge). In addition, we consider van der Waals heterostructures (vdW) of Janus type S-Mo-SiN${}_{2}$ with graphene and estimate their basic properties.

## 2. Optimization and Stability of Structures

Presented computational results were obtained within the density functional theory (DFT) implemented in the Quantum ATK package [14]. To optimize the initial structures and to relax materials under stress, we have chosen pseudopotential PseudoDojo [15] with a linear combination of atomic orbitals (LCAO). The exchange–correlation potential was described by the generalized Perdew–Burke–Ernzerhof gradient approximation (GGA-PBE) [16]. The 15 × 15 × 1 grid in the first Brilouin zone was used for optimization.

The structures were optimized until the maximum force acting on each atom becomes less than 0.01 eV/Å, and the maximum energy change between two stages becomes less than 10${}^{-5}$ eV. To avoid the influence of boundary conditions in the direction perpendicular to the 2D sheet, the lattice cell parameter 30 Å is used. The band structures, optical properties, and spin-polarized characteristics were calculated using the Heyd–Scuseria–Ernzerhof (HSE06) hybrid exchange–correlation functional. The 25 × 25 × 1 grid in the first Brillouin zone was used to calculate the electronic and optical properties.

The formation energy per atom is calculated according to the following formula:

$$E_{\mathrm{form}}=\frac{E_{\mathrm{total}}-E\left(M\;=\;\mathrm{Mo},\;W\right)-E\left(A\;=\;\mathrm{Si},\;\mathrm{Ge}\right)-E\left(X\;=\;S,\;\mathrm{Se},\;\mathrm{Te}\right)-2E\left(N\right)}{N},$$

where $E_{\mathrm{total}}$ is the total energy of cluster, *E* are energies of elements calculated for optimized Mo,W, α−Si, α−Ge, orthorhombic sulfur, γ−Se, α−Te, and molecules $N_{2}$; *N* is the number of atoms in the translated cluster (unit cell).

Figure 1 shows the structure of studied X-M-AN${}_{2}$ layers and indicates the main geometric parameters. Calculated values of these parameters are listed in Table 1. The results of calculation for the negative formation energy are also given there.

All proposed structures have a negative formation energy, which means that they should be stable. Furthermore, we limit our consideration on the following most stable structures: SWSiN${}_{2}$, SeWSiN${}_{2}$, TeWSiN${}_{2}$, and SWGeN${}_{2}$.

**Figure 2:**
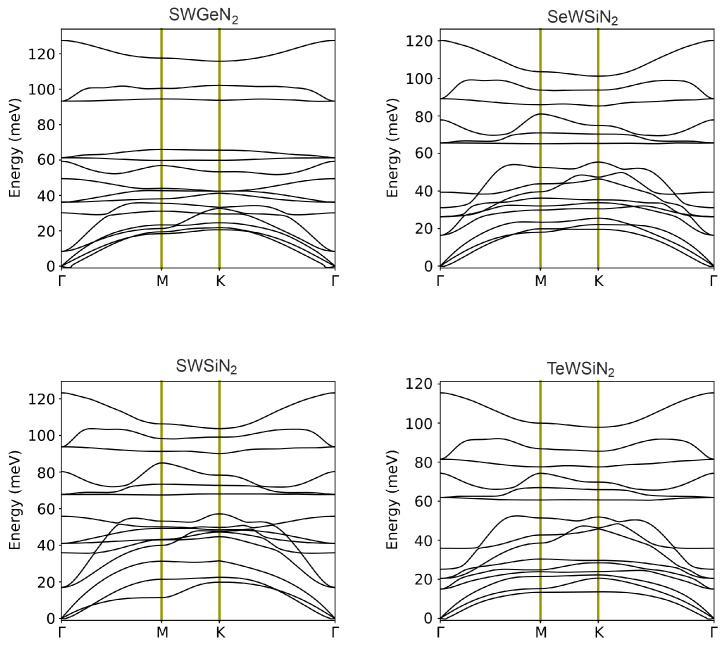
Phonon spectra of X-M-AN${}_{2}$ monolayers. The three lower branches correspond to acoustic modes TA, LA, and ZA, and the remaining 12 branches represent optical modes. The intersection of the optical branches at the Γ point indicates their degeneracy.

In equilibrium, the potential energy is minimal, and it increases with small displacements of atoms from the positions of stable configuration. This fact makes it possible to use vibrational spectra as a criterion for the stability of materials [17]. To estimate the dynamic stability of optimized structures, the phonon dispersion characteristics of monolayers were calculated (Figure 2). The elementary cell contains five atoms, so its phonon spectrum consists of 15 phonon branches, 3 acoustic and 12 optical. Three acoustic branches have a lower frequency and contain in-plane longitudinal (LA), transverse (TA) and out-of-plane (ZA) acoustic modes. There are no imaginary frequencies in the Brillouin zone, except for small fragments near the Γ point formed by the ZA mode. The maximum values of these frequencies are 0.165, 0.285, 0.141, and 0.771 meV (or 1.33, 2.30, 1.41, and 6.22 cm${}^{-1}$) for SeWSiN${}_{2}$, TeWSiN${}_{2}$, SWSiN${}_{2}$, and SWGeN${}_{2}$, respectively. This slight instability is due to the difficulty of achieving numerical convergence for the flexural acoustic phonons (ZA) branch when using first principles calculations for 2D materials. In addition, even higher imaginary frequencies of 14 and 280 cm${}^{-1}$ were reported for 1T${}^{\prime }$-MY${}_{2}$ monolayers (M = Mo, W; Y = S, Se, Te) [18] and 1T-MoS${}_{2}$ [19], respectively.

Twelve optical modes comprise 4 in-plane longitudinal (LO), 4 in-plane transverse (TO), and 4 out-of-plane (ZO) optical modes. Based on irreducible representations at point Γ, these modes are classified as doubly degenerate *E* (LO and TO) non-degenerate $A_{1}$ (ZO). There is no frequency gap between the acoustic and optical branches, and the conservation law for energy in phonon–phonon scattering is easy to fulfill, which leads to a high phonon scattering rate. In particular, the process of annihilation of two acoustic modes into one optical mode becomes highly efficient.

## 3. Electronic Properties of Structures

**Figure 3:**
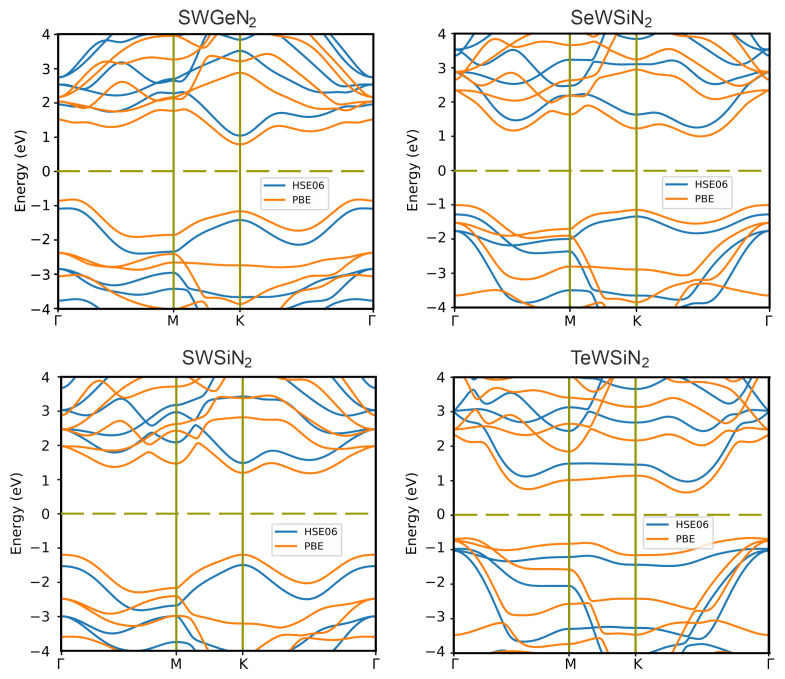
Band structures of 2D XMoSiN${}_{2}$ (X = S, Se, Te) calculated using PBE (orange lines) and HSE06 (blue lines) functionals. The dashed line represents the Fermi level.

Figure 3 demonstrates band structures obtained using PBE and HSE06 exchange–correlation functionals. The calculation results with HSE06 show that SWSiN${}_{2}$ is a direct-gap semiconductor with the conduction band minimum (CBM) and valence band maximum (VBM) located at the same point K. Applying PBE to this structure makes it indirect-gap and changes the transition to Γ→ K. The CBM and VBM of the remaining monolayers lie at different points, giving rise to indirect band structures for both functionals. Table 2 lists the bandgap values for considered monolayers. The value of E${}_{g}^{\mathrm{PBE}}$ is expectedly less than E${}_{g}^{\mathrm{HSE}06}$. In further calculations, the HSE06 functional is used because for bandgaps, and it gives smaller errors than PBE [20].

**Figure 4:**
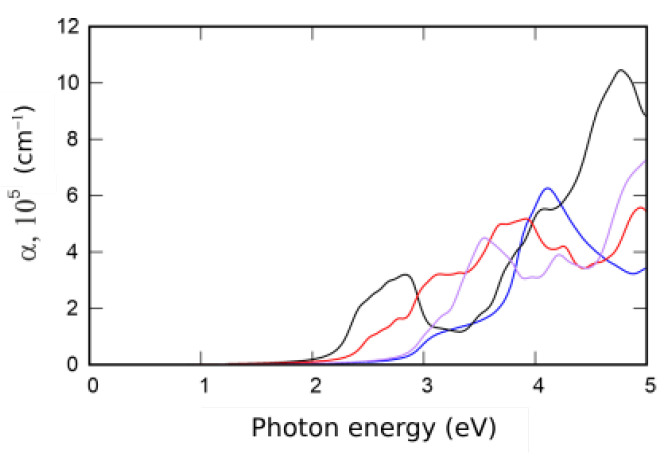
Absorption coefficient of XMAN${}_{2}$: SWSiN${}_{2}$ (blue line), TeWSiN${}_{2}$ (black line), SWGeN${}_{2}$ (red line), and SeWSiN${}_{2}$ (purple line).

Absorption spectra of XMAN${}_{2}$ monolayers are presented in Figure 4. The used computational approach is based on the Kubo–Greenwood formalism and described particularly in [9,21].

**Figure 5:**
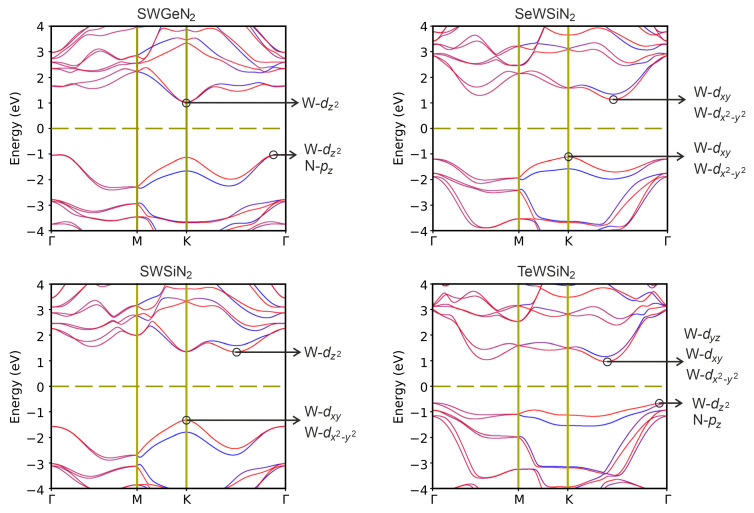
Band structures XMoSiN${}_{2}$ (X = S, Se, Te) calculated using HSE06+SOC. Inserts indicate the states forming VBM and CBM.

In the structures under study, spin–orbit splitting (SOC) can be observed due to the absence of a horizontal reflection plane. In Figure 5, the projected bandstructures calculated taking into account the SOC interaction are presented. It can be seen that the interaction reduces the bandgap. In addition, for SeWSiN${}_{2}$, SOC shifts VBM from the valley K →Γ to point K, and SWSiN${}_{2}$ becomes an indirect-gap semiconductor.

In Figure 5, one can find that the electronic states near CBM and VBM are mainly associated with the *d*-orbitals of the tungsten atom, and only VBM of SWGeN${}_{2}$ and TeWSiN${}_{2}$ are also partially represented by N-p${}_{z}$ states.

Strong hybridization between the W-$d_{x^{2}-y^{2}}$ and W-$d_{xy}$ orbitals results in a large SOC splitting. For all structures, the maximum splitting of the valence band is found at the K point, while for the conduction band in the K→Γ and Γ→M valleys splitting values of the valence and conduction bands.

**Figure 6:**
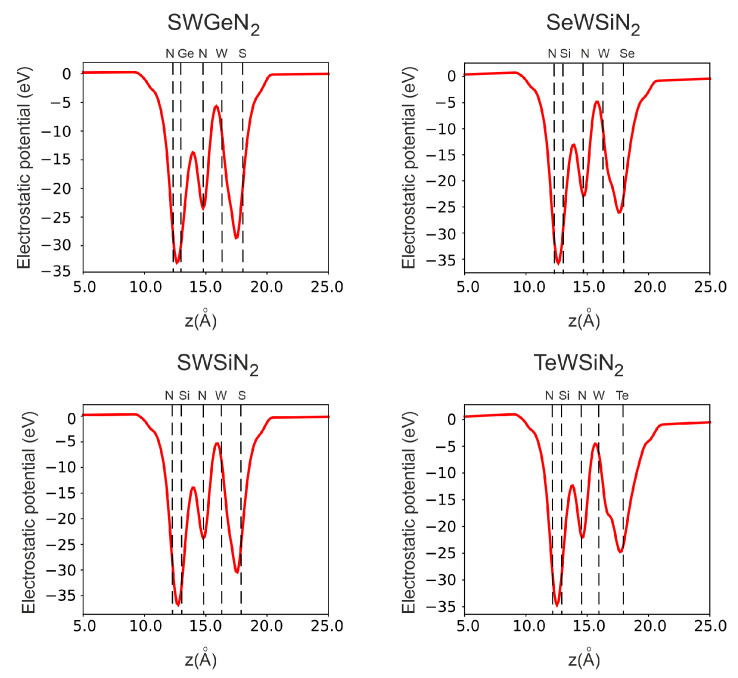
Electrostatic potential in X-W-AN${}_{2}$ (X = S, Se, Te, A = Si, Ge). Dashed lines indicate the positions of atoms.

**Figure 7:**
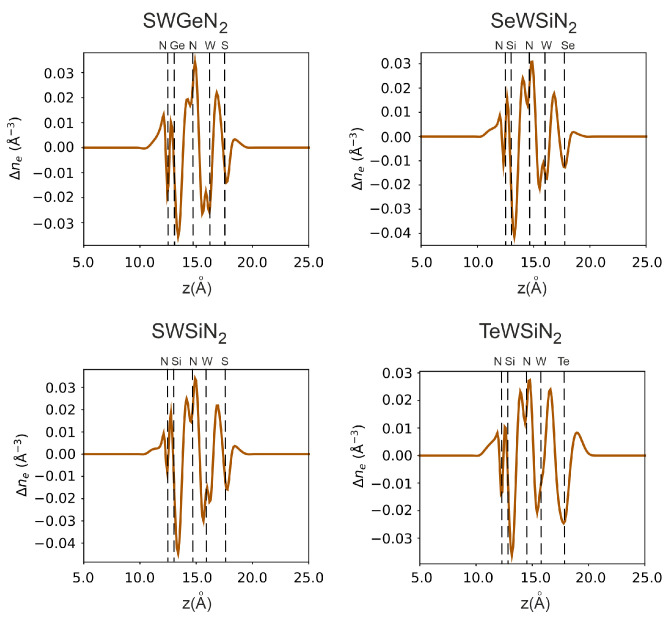
Monolayer electron difference density in X-W-AN${}_{2}$ (X = S, Se, Te, A = Si, Ge).

In Figure 6 and Figure 7, we additionally investigate vertical polarization by plotting the electrostatic potential and electron density difference along the *z*-axis. All structures have an electrostatic potential difference Δϕ, with the potential being higher on the side of the external nitrogen atom. In addition, there is a redistribution of electrons. The central nitrogen atom, due to its high electronegativity (3.04), pulls electrons from neighboring tungsten, silicon, or germanium atoms for SWGeN${}_{2}$ (the electronegativity values? are 2.36, 1.9, and 2.01, respectively). Moreover, from the side of W, $\Delta n_{e}$ is larger than from the side of Si or Ge because tungsten has more electrons. The outer nitrogen atom also pulls electrons from silicon, but to a lesser extent than the central one. The electronegativity of the external chalcogens Se and S (2.55 and 2.58, respectively) is slightly higher than the electronegativity of W, so the maximum electron concentration is at the interface between tungsten and selenium or sulfur and is slightly shifted to the latter, in contrast to Te (2.10), where the electrons are concentrated, on the contrary, closer to the metal. Charge depletion is observed on the chalcogen atoms themselves. Due to the redistribution of electrons, local electric fields arise, one directed from the outer nitrogen atom to the central one, and the other from the chalcogenide atom to the central N. Thus, a resulting field arises directed from the chalcogen atom to the outer nitrogen. The largest intensity of this built-in field is in SeWSiN${}_{2}$ 1.91 VÅ${}^{-1}$, for TeWSiN${}_{2}$ it is 1.86 VÅ${}^{-1}$, for SWSiN${}_{2}$–1.31 VÅ${}^{-1}$, and for SWGeN${}_{2}$–0.90 VÅ${}^{-1}$. This indicates a stronger vertical polarization of the SeWSiN${}_{2}$ monolayer. A positive charge at the boundary of the structure (Se, S, and Te atoms) facilitates the escape of electrons, which leads to a decrease in the work function and the potential energy difference of 2.01 eV, 1.61 eV, and 0.47 eV for TeMoSiN${}_{2}$, SeMoSiN${}_{2}$, and SMoSiN${}_{2}$, respectively.

The built-in field promotes charge separation, which makes these structures promising for photocatalysis and optoelectronics. The positive charge at the boundary (Se, S, and Te atoms) facilitates the escape of electrons that leads to a decrease in the work function and the potential energy difference. In addition, a dipole moment μ proportional to Δϕ arises. Due to the large electronegativity difference between Te and nitrogen, TeWSiN${}_{2}$ has the largest dipole moment (Table 2).

**Figure 8:**
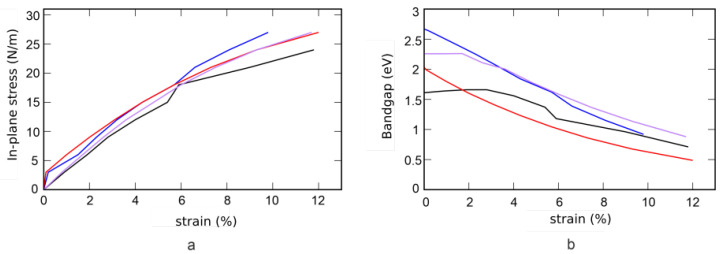
Dependences of stress (**a**) and band gap (**b**) on strain. SWSiN${}_{2}$ (blue line), TeWSiN${}_{2}$ (black line), SWGeN${}_{2}$ (red line), and SeWSiN${}_{2}$ (purple line).

One way to change the bandgap is to stretch the monolayer in the plane. Figure 8 shows the stress (a) and bandgap (b) versus strain. In addition, elastic moduli, Young’s modulus *Y*, and Poisson’s ratio ν are listed in Table 3. Deviation from the linear Hooke’s law takes place at deformations greater than 0.1% for SWGeN${}_{2}$ and SeWSiN${}_{2}$, for SWSiN${}_{2}$ and TeWSiN${}_{2}$ deviation from linearity is observed at deformations exceeding 3 % (Figure 8a). Due to the higher Young’s modulus, SeWSiN${}_{2}$ stretches worse than other structures, and when it reaches 10% deformation, it fractures. For comparison, the values ??of elastic moduli for graphene and MoS${}_{2}$ are also given. Young’s modulus XWSiN${}_{2}$ is close to the value of graphene and is 2.5 times higher than that of two-dimensional molybdenite. Poisson’s ratio of SWGeN${}_{2}$ is close to values of TMDs.

Deformation of the structure in the plane changes the bandgap. The gap of SWSiN${}_{2}$ and TeWSiN${}_{2}$ changes irregularly with deformation: at first, it slightly increases and then decreases. SWGeN${}_{2}$ and SeWSiN${}_{2}$ behave more predictably. The linear decrease in the SeWSiN${}_{2}$ gap to a strain of 6% makes this material promising for use in flexible electronics.

## 4. Van Der Waals Heterostructures with Graphene

Van der Waals heterostructures based on 2D layered materials with selected properties pave the way for integration at the atomic level and can lead to new heterostructures with completely new physics and versatility. Janus 2D materials with different surfaces have attracted intensive research interest due to the remarkable properties induced by symmetry breaking. In recent times, various vdW heterostructures with Janus TMDCs have been studied [22,23,24]. In our recent works [25,26], we proposed and studied novel vdW heterostructures obtained by stacking of graphenylene with TMDCs and Janus XMoY (X ≠ Y = S, Se, Te) monolayers.

In [27], the authors studied vdW heterostructures consisting of MoSi${}_{2}$N${}_{4}$ in contact with graphene and NbS${}_{2}$ monolayers using the density functional theory calculations. They have shown that the MoSi${}_{2}$N${}_{4}$/NbS${}_{2}$ contact exhibits an ultra-low Schottky barrier height, which is beneficial for nanoelectronics’ applications. For the MoSi${}_{2}$N${}_{4}$/graphene contact, the Schottky barrier height can be modulated with interlayer spacing or with external electric fields. The maximum of absorption spectrum falls at a wavelength of 510 nm. The tube is characterized by significant optical absorption in the entire optical range.

**Figure 9:**
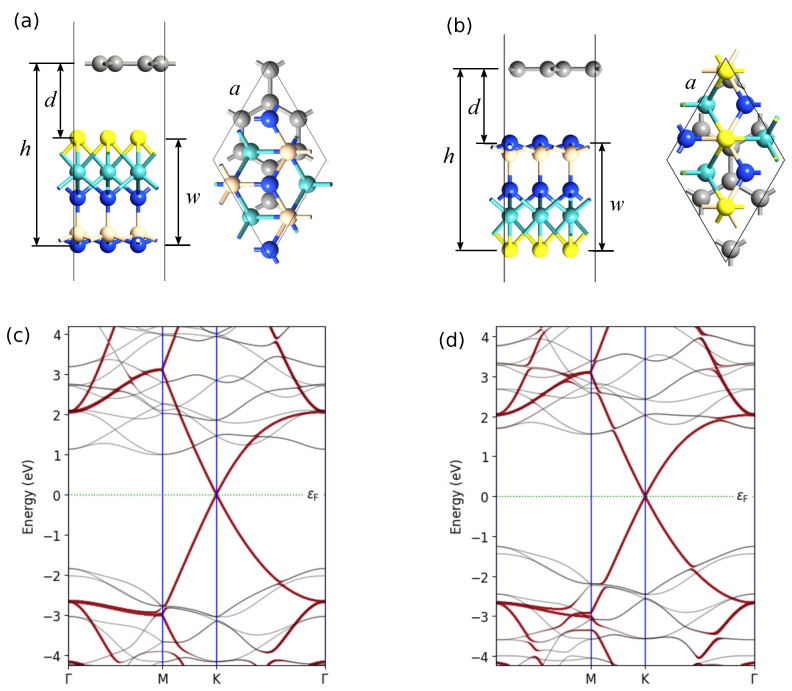
Geometry of vdW heterostructure S-MoSiN${}_{2}$/graphene and graphene/S-MoSiN${}_{2}$ (**a**,**b**) and their bandstructures (**c**,**d**). The brown lines correspond to the contribution of carbon.

**Figure 10:**
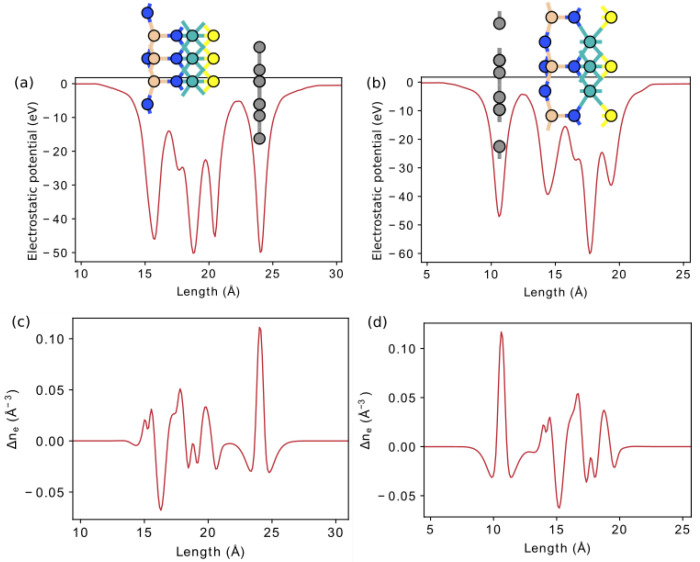
Electrostatic potential and electron difference density for vdW heterostructure S-MoSiN${}_{2}$/graphene (**a**,**c**) and graphene/S-MoSiN${}_{2}$ (**b**,**d**).

Apparently, it is possible to form a van der Waals contact of the Janus layers considered here with graphene and two-dimensional dichalcogenides. We consider structures with graphene in more detail (Figure 9). Table 4 provides information about geometric parameters of optimized vdW heterostructures graphene/S-MoSiN${}_{2}$ and S-MoSiN${}_{2}$/graphene. The optimization was performed with exchange–correlation potential described by PBE GGA [16], with vdW corrections incorporated with Grimme’s DFT-D2 method [28]. The structure optimization is carried out until the maximum force on each atom becomes less than 0.01 eV/ Å, and the maximum energy change between the two steps is smaller than 10${}^{-5}$ eV. In both structures, the graphene was stretched (ε≈2.8 %), while the Janus monolayer S-MoSiN${}_{2}$ was compressed (ε≈−1.9 %) after optimization of vdW junction. Electrostatic potential and electron difference density for these vdW heterostructures are plotted in Figure 10.

**Figure 11:**
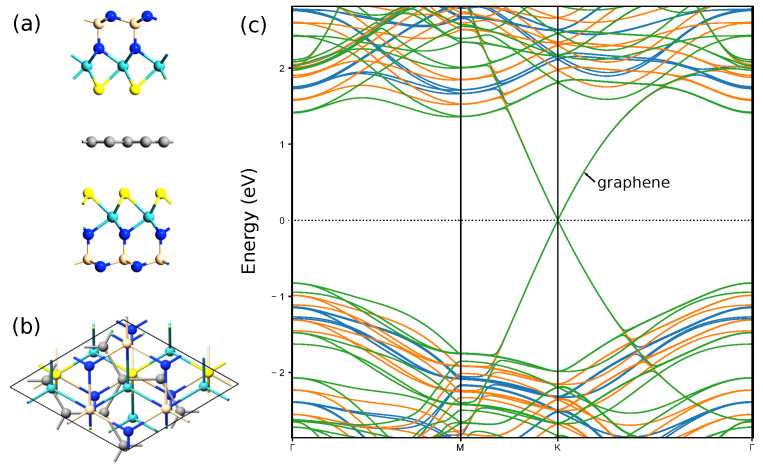
Geometry of three-layer vdW heterostructure S-MoSiN${}_{2}$/graphene/S-MoSiN${}_{2}$ (**a**,**b**). Bandstructures (**c**) are calculated for three values of electric field: 0 V/nm (blue line), 0.667 V/nm (orange line), and 1.333 V/nm (green line).

A large built-in transverse electric field in Janus X-M-ZN${}_{2}$ monolayers can be utilized in photovoltaic applications. Also, we show that these monolayers can serve as shielding layers. In Figure 11, geometry of three-layer vdW junction S-MoSiN${}_{2}$/graphene/S-MoSiN${}_{2}$ is shown. Calculated bandstructures corresponding to three values of electric field: 0 V/nm, 0.667 V/nm, and 1.333 V/nm indicate that the branches corresponding to the graphene contribution are insensitive to an external transverse electric field in such a structure. In this case, the branches corresponding to the Janus layers shift significantly.

## 5. Conclusions

In our recent work [9], for the first time, we proposed and theoretically investigated the asymmetric 2D materials XMoSiN${}_{2}$ (X = S, Se, Te), constructed and optimized from MoSi${}_{2}$N${}_{4}$. In this work, we continue studying other representatives of the Janus type monolayers X-M-ZN${}_{2}$ (X = S, Se, Te; M = Mo, W; A = Si, Ge). These monolayers were constructed and optimized from MoSi${}_{2}$N${}_{4}$ by replacing SiN${}_{2}$ on one side with chalcogen atoms (sulfur, selenium, or tellurium), and possibly replacing molybdenum (Mo) to tungsten (W) and/or silicon (Si) to germanium (Ge). The new structure is a hybrid of a transition metal dichalcogenide and a 2D material from the MoSi${}_{2}$N${}_{4}$ family. The stability of new 2D materials has been substantiated by means of DFT-based calculations. The monolayers under study are characterized by high values of binding energy >7.5 eV/atom. First-principle studies of several representatives, such as MTeSiX_2_ (M = Mo, W; X = N, P, As) monolayers, were studied in [13]. In all studied monolayers, a large built-in transverse electric field arises due to the redistribution of electrons, enabling separation of the generated electron-hole pairs within one layer that is promising for photovoltaic applications. Possible vdW heterostructures of Janus type monolayers with graphene are estimated. It is particularly shown that the Janus type MoSSiN${}_{2}$ monolayers effectively shield a constant external electric field.

## Figures and Tables

**Table 1:** Lattice parameter *a*, bond lengths $l_{1}$, $l_{2}$, $l_{3}$, $l_{4}$ and thickness *h* of optimized Janus structures X-M-AN${}_{2}$ (X = S, Se, Te; M = Mo, W, A = Si, Ge) and formation energy $E_{\mathrm{form}}$.

	*a*, Å	$l_{1}$, Å	$l_{2}$, Å	$l_{3}$, Å	$l_{4}$, Å	*h*, Å	$E_{\mathrm{form}}$, eV/atom
SWSiN${}_{2}$	2.999	2.39	2.12	1.77	1.80	5.13	−0.93
SeWSiN${}_{2}$	3.042	2.55	2.13	1.77	1.82	5.29	−0.70
TeWSiN${}_{2}$	3.113	2.72	2.15	1.77	1.85	5.44	−0.37
SWGeN${}_{2}$	3.105	2.47	2.14	1.77	1.85	5.08	−0.38
SeWGeN${}_{2}$	3.132	2.56	2.15	1.91	1.90	5.47	−0.26
TeWGeN${}_{2}$	3.105	2.72	2.14	1.77	1.85	5.43	−0.12
SMoGeN${}_{2}$	3.127	2.27	2.14	1.77	1.86	4.67	−0.21
SeMoGeN${}_{2}$	3.125	2.54	2.14	1.90	1.90	5.44	−0.33
TeMoGeN${}_{2}$	3.193	2.72	2.16	1.91	1.93	5.60	−0.06
SMoSiN${}_{2}$ [9]	2.989	2.38	2.11	1.76	1.80	5.12	−0.96
SeMoSiN${}_{2}$ [9]	3.034	2.53	2.12	1.77	1.83	5.27	−0.77
TeMoSiN${}_{2}$ [9]	3.105	2.72	2.14	1.77	1.85	5.43	−0.46

**Table 2:** Bandgap $E_{g}$ calculated using PBE, HSE06, and HSE06+SOC functionals, SOC splitting $\Delta_{V}$ for valence band maximum and $\Delta_{C}$ for conduction band minimum, potential energy difference δϕ and dipole moment μ in Janus structures X-W-AN${}_{2}$ (X = S, Se, Te; A = Si, Ge).

	E${}_{g}^{\mathrm{PBE}}$, eV	E${}_{g}^{\mathrm{HSE}06}$, eV	E${}_{g}^{\mathrm{HSE}06+\mathrm{SOC}}$, eV	$\Delta_{V}$, meV	$\Delta_{C}$, meV	Δϕ, eV	μ, D
SWSiN${}_{2}$	2.37	2.98	2.67	475	254	0.65	0.238
SeWSiN${}_{2}$	2.01	2.54	2.26	329	454	1.60	0.403
TeWSiN${}_{2}$	1.32	1.95	1.62	410	406	2.05	0.530
SWGeN${}_{2}$	1.61	2.12	2.03	523	219	0.42	0.224

**Table 3:** Elasticity moduli, Young’s modulus *Y*, Poisson’s ratio ν for Janus monolayers X-W-AN${}_{2}$ (X = S, Se, Te, A = Si, Ge), compared with those for graphene and MoS2.

	$c_{11}$, N/m	$c_{12}$, N/m	$c_{66}$, N/m	*Y*, N/m	ν
SWSiN${}_{2}$	339.43	74.59	131.88	323.04	0.22
SeWSiN${}_{2}$	339.47	69.79	130.83	325.12	0.21
TeWSiN${}_{2}$	312.24	56.95	130.34	301.85	0.18
SWGeN${}_{2}$	274.17	68.64	100.68	256.99	0.25
MoS${}_{2}$	128.40	32.60	–	120.10	0.25
graphene	352.00	62.60	–	340.80	0.18

**Table 4:** Geometric parameters of vdW heterostructures graphene/S-MoSiN${}_{2}$ and S-MoSiN${}_{2}$/graphene.

	*h*, Å	*d*, Å	*w*, Å	*a*, Å
graphene/S-MoSiN${}_{2}$	8.771	3.597	5.1739	5.080
S-MoSiN${}_{2}$/graphene	8.736	3.564	5.1725	5.081

## Data Availability

Not applicable.
